# Women’s experiences of counselling in cases of a screen-positive prenatal screening result

**DOI:** 10.1371/journal.pone.0247164

**Published:** 2021-03-10

**Authors:** Leena Vuorenlehto, Kaisa Hinnelä, Outi Äyräs, Veli-Matti Ulander, Pekka Louhiala, Marja Kaijomaa

**Affiliations:** 1 Department of Obstetrics and Gynecology, Helsinki University Hospital and University of Helsinki, Helsinki, Finland; 2 Department of Public Health, University of Helsinki, Helsinki, Finland; Universita degli Studi di Roma Tor Vergata, ITALY

## Abstract

**Objective:**

To study women’s apprehensions, understanding and experiences of counselling concerning a screen-positive result in screening for fetal chromosomal defects.

**Methods:**

A questionnaire study including different steps of the prenatal screening process was carried out in Helsinki University Hospital. Women’s experiences concerning counselling immediately after a screen-positive result and during further examinations in the Fetal Medicine Unit (FMU) were analyzed.

**Results:**

143 women filled in the questionnaire. Less than half of the women considered the primary counselling after a screen-positive result to be explicit (43.9%) and sufficient (43.1%). In the FMU, 88.3% and 89.8% of women were satisfied with the explicitness and sufficiency of counselling. Most women (75%) experienced worry before further examinations but less than half (45%) had considered their personal values concerning diagnostic tests. Half (50.5%) of women expected the worry to continue even if diagnostic tests turn out normal. Most (81%) women were aware that diagnostic tests are voluntary and were confident (85.3%) with their decision to participate.

**Conclusions:**

After a screen-positive result, women have unanswered questions, experience anxiety and confusion. The possibility of an abnormal screening result is not seriously considered beforehand. To enable an informed consent for prenatal screening, improvements in prescreening counselling during the first visits of antenatal care need to be made.

## Introduction

Prenatal screening is part of the public health care system in Finland and available to everyone. It is regulated by the Governmental Decree of Screening (Health Care Act 339/2011) and organized by local authorities. Screening is free of charge and voluntary [1].

The main goal of prenatal screening is to identify chromosomal and structural abnormalities in the first and second trimester of pregnancy [2]. Screening increases families’ autonomy and decreases infant morbidity and mortality.

In Finland, community-based maternal care units provide free maternal care services and are responsible for informing women about prenatal screening and screening options. During the first antenatal visit, women receive oral and written information about prenatal screening and after familiarizing themselves with it, they are expected to give an informed consent concerning participation or refusal. An informed consent is defined by Marteau et al, [3] as a choice that is “based on relevant knowledge, consistent with the decision-maker’s values and behaviorally implemented”. In Finland most women choose to participate.

Prenatal screening is performed in steps in the first and second trimester. The screening for chromosomal abnormalities is based on the measurement of fetal nuchal translucency (NT) at 11+0–13+6 gestational weeks (gw). It is often combined with the measurement of maternal serum pregnancy associated placental protein-A (PAPP-A) and human chorionic gonadotropin (hCG) at 9+0–11+6 gw to form the first trimester combined screening (FTS). It is the most popular test in screening for chromosomal abnormalities and with a false positive rate (FPR) of 3–5%, the detection rate for Down syndrome (DS) is usually 80–85%, but has reached 90% [4, 5]. The majority of Finnish women choose to participate in the FTS.

In cases of an incorrect dating of pregnancy or a missed time frame for the FTS, a serum screening consisting serum alpha-fetoprotein and hCG can also be performed at 15+0–16+6 gw. The detection rate for chromosomal abnormalities is lower than in the FTS. In the FTS and the second trimester serum screening, a risk cut-off 1/250 is used to indicate an increased risk for DS.

**Fig 1:**
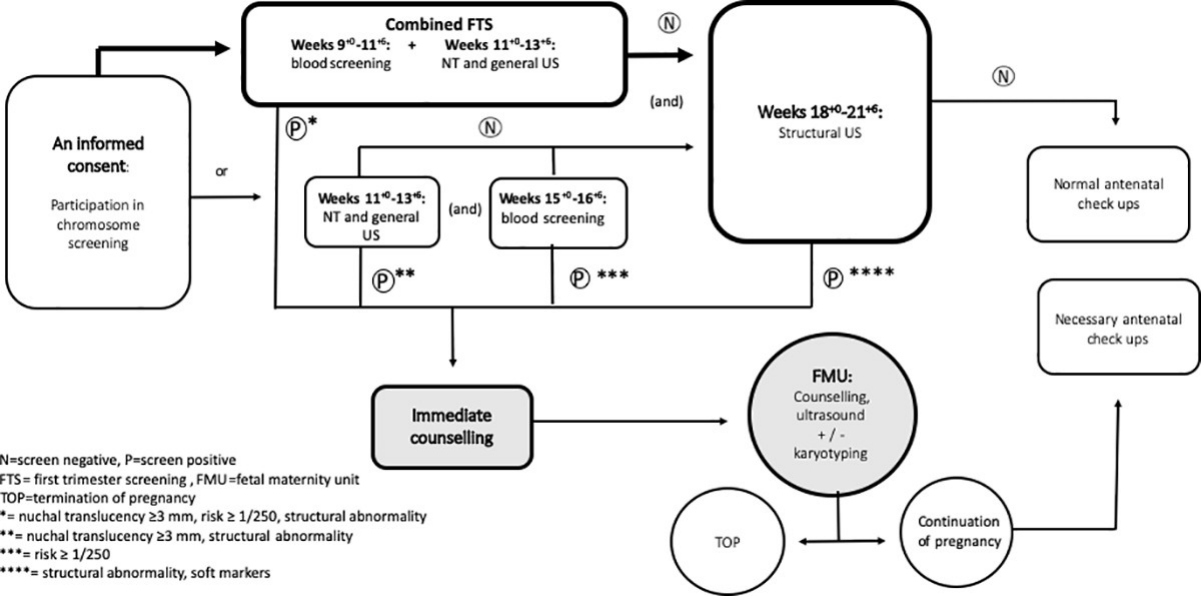
Prenatal screening in Finland.

If opted for, the FTS or second trimester serum screening is followed by the second trimester general ultrasound (US) at 18+0–21+6 gw to detect major structural abnormalities or other signs of chromosomal abnormality. Most women participate in the FTS and second trimester general US. (Fig 1). In the majority of pregnancies, the first and second trimester US are performed by trained midwives.

In cases of an abnormal finding in the first or second trimester US, immediate counselling is given by the midwife or a senior consultant, a specialist in fetal medicine. In cases of an abnormal FTS or second trimester serum screening result, a phone call concerning the result by a trained midwife is given. In both cases, detailed US with diagnostic tests in the FMU are offered.

In Finland the termination of pregnancy (TOP) for social indications is allowed up to 12+0 gw and in cases of exceptional social indications, suspected or diagnosed severe fetal defect, illness or handicap, up to 20+0 gw. In cases of diagnosed severe fetal illness, TOP is allowed up to 24+0 gw [6].

The process of prenatal screening has been shown to cause anxiety, especially in cases with an increased risk for DS [7–9]. The anxiety may also continue even after receiving a normal result from further diagnostic tests [7, 10]. It may also have long-lasting effects on prenatal attachment and the postnatal mother-child relationship. An increased risk of preterm delivery, reduced birth weight and effects on toddlers’ development have also been described [8, 11, 12].

Earlier studies have shown that some women find the pre-screening information inadequate, and some are not aware that the main purpose of the screening is to find abnormalities [7, 13, 14]. Women are expected to give their consent in all steps of screening, but that may be impossible due to the lack of sufficient counselling. This could be linked to increased anxiety, as sufficient information about screening and an informed consent have been shown to reduce pre-screening anxiety [11].

The main purpose of this study was to establish women’s experiences during the screening process and to find out how the objectives concerning sufficient counselling are met in Helsinki University Hospital (HUH). As part of a larger study, this article focuses on women with a screen-positive result, their experiences on the counselling and feelings of anxiety. These themes are covered in order to determine how the screening process can be improved in the future.

## Material and methods

A large questionnaire study concerning information, understanding and attitudes of women attending prenatal screening was carried out in HUH Women’s Clinic. The study period was between August 1, 2014 and February 28, 2015. Women were enrolled by the medical staff when arriving to the first trimester US and written information concerning the study was given. Questionnaires were then offered before and after the first and second trimester US, and possible appointments in the FMU. The questionnaire was pilot tested by patients before the study and questions were available in both official languages (Finnish and Swedish).

In this study we focused on women who received a screen-positive result at any point of the screening process. In cases of an increased NT in the first trimester US, the primary counselling was given immediately during the appointment by a trained midwife. In addition, written information concerning chromosomal abnormalities and invasive karyotyping was given. In cases of other abnormalities in the first or second trimester US, a specialist in fetal medicine was consulted by a trained midwife and primary counselling and verbal information concerning the abnormality was given during the appointment. Women with an increased risk in FTS, were informed about the result by telephone and counselling was given by a trained midwife. Women were advised to search the written information concerning chromosomal abnormalities and invasive karyotyping on the hospital website.

**Fig 2:**
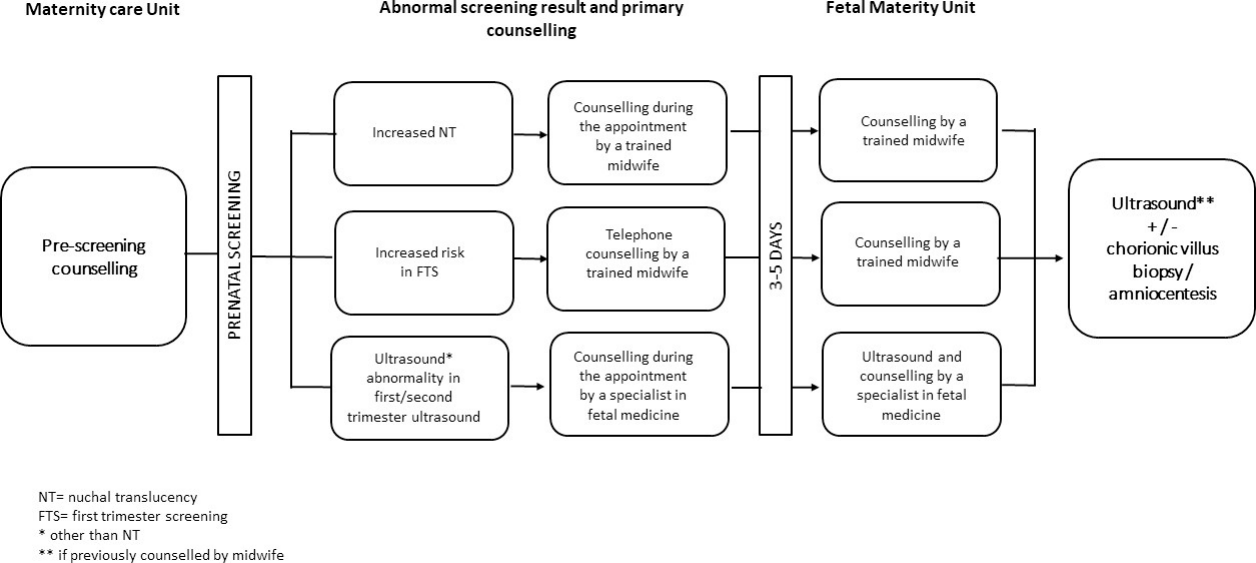
The protocol of prenatal screening.

In all cases of a screen-positive result, women were offered an appointment in the FMU of HUH. In cases of an increased NT or an increased risk in the FTS, counselling by a trained midwife was first given to make an informed consent concerning invasive testing. In other cases, a detailed US and counselling was performed by a specialist in fetal medicine. Fetal karyotyping by either chorionic villus sampling or amniocentesis was offered to all women as a diagnostic chromosomal test and they were informed about the 0.5–1.0% risk of miscarriage. Non-invasive prenatal test (NIPT) was not yet routinely used in Helsinki University Hospital at the time of the study (Fig 2).

All Finnish or Swedish speaking women attending the FMU were asked to participate in the study and to fill in a questionnaire before and after their appointment. Participation was voluntary and all women were informed about the aims of the study. After information, an informed verbal consent was given by all women and returning of the filled in questionnaire was considered as a decision to participate. Women were informed that their decision regarding participation did not impact their treatment.

To examine women’s feelings and their experiences concerning the previously given counselling, the following questions were asked before the appointment in the FMU.

How did you experience

- the explicitness of primary counselling?
- the sufficiency of primary counselling?

What information did you miss?

The questionnaire also included questions about the feelings of worry and anxiety:

- How worried do you feel about the results of the upcoming examination?
- Did you consider your personal values beforehand and weigh different options in case of a screen-positive result?

These questions were answered by choosing a score from one to five in a range where number one represented the worst/lowest score and number five the best/highest (very poor/very little–very good/very much).

Women were also asked whether they were informed about the voluntariness of further examinations and whether personal consideration of participation was given. These questions were answered Yes or No.

- Were you told that further examinations are voluntary?
- Did you at any point consider refusing further examinations?

After the appointment in the FMU, the following questions concerning counselling, examination indications and findings in the FMU were asked. These questions were answered by a score from one (very poor/very little) to five (very good/very much).

How did you experience

- the explicitness of counselling?
- the sufficiency of counselling?
- Are you familiar with the terminology used in prenatal screening (chromosome, gene, nuchal translucency, structural abnormality)? Are these terms easy to understand?
- Did you understand the indication to the appointment/examinations in the FMU?
- Did you understand the findings of the examination? Was the information clear?
- Does your worry and anxiety concerning your pregnancy increase, even if the diagnostic tests (amniocentesis/chorionic villus sample) turn out normal?

An open question was available to describe feelings not specifically asked in the questionnaire.

Statistical analysis of answers was performed using SPSS version 22 software (IBM Corporation, Armonk, NY) and the permission for this study was obtained from Research Ethics Committee of Helsinki and Uusimaa hospital district (decision 60/13/03/03/2014).The Wilcoxon Signed Ranked test was used to analyze the answers of same women before and after the appointment in the FMU.

## Results

After a screen-positive result, 143 women attending the FMU took part in the study. This represents more than 80% of eligible women attending FMU during the study period. Women attending FMU with other indications (an abnormality in previous pregnancy, a hereditary abnormality) were excluded from the study.

**Fig 3:**
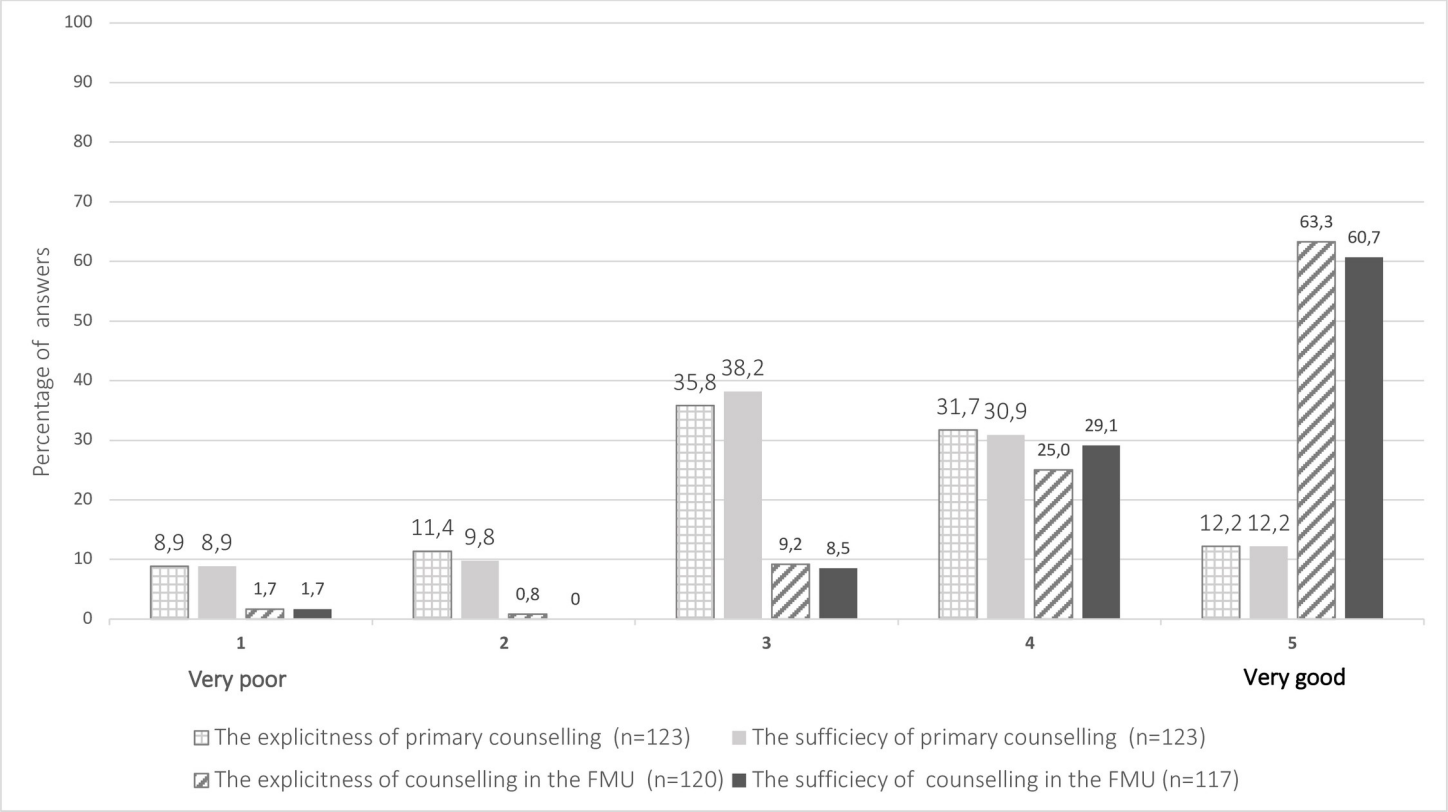
The explicitness and sufficiency of counselling.

The mean age of mothers was 33.5 (± 5.8) years and 30.8% (44/143) were primigravidas. A significant difference in the explicitness and sufficiency of counselling was detected between the primary counselling and counselling in the FMU. The mean value of the explicitness of the primary counselling (3.43) was significantly lower than the mean value of counselling in the FMU (4.48) (*p*<0.001). The mean values of the sufficiency of counselling were 3.28 and 4.47 respectively (*p*<0.001). Less than half of the women (43.9%) found the immediate counselling to be explicit enough (4–5), while the majority (88.3%) were satisfied with the counselling given in the FMU. Also, less than half (43.1%) of the women found the immediate counselling sufficient, compared to the majority (89.8%) at the appointment in the FMU (Fig 3).

After the screen-positive result, 25.7% of the respondents answered the open question about the information lacking before the appointment in the FMU. Many wished for more information about the factors behind their increased risk for chromosomal abnormality and some wished for more details regarding karyotyping. Confusion and anxiety were common.

**Fig 4:**
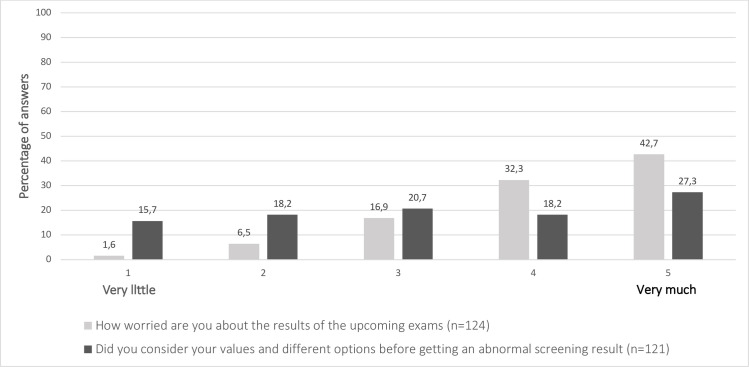
The experienced worry and readiness concerning an abnormal screening result.

**Fig 5:**
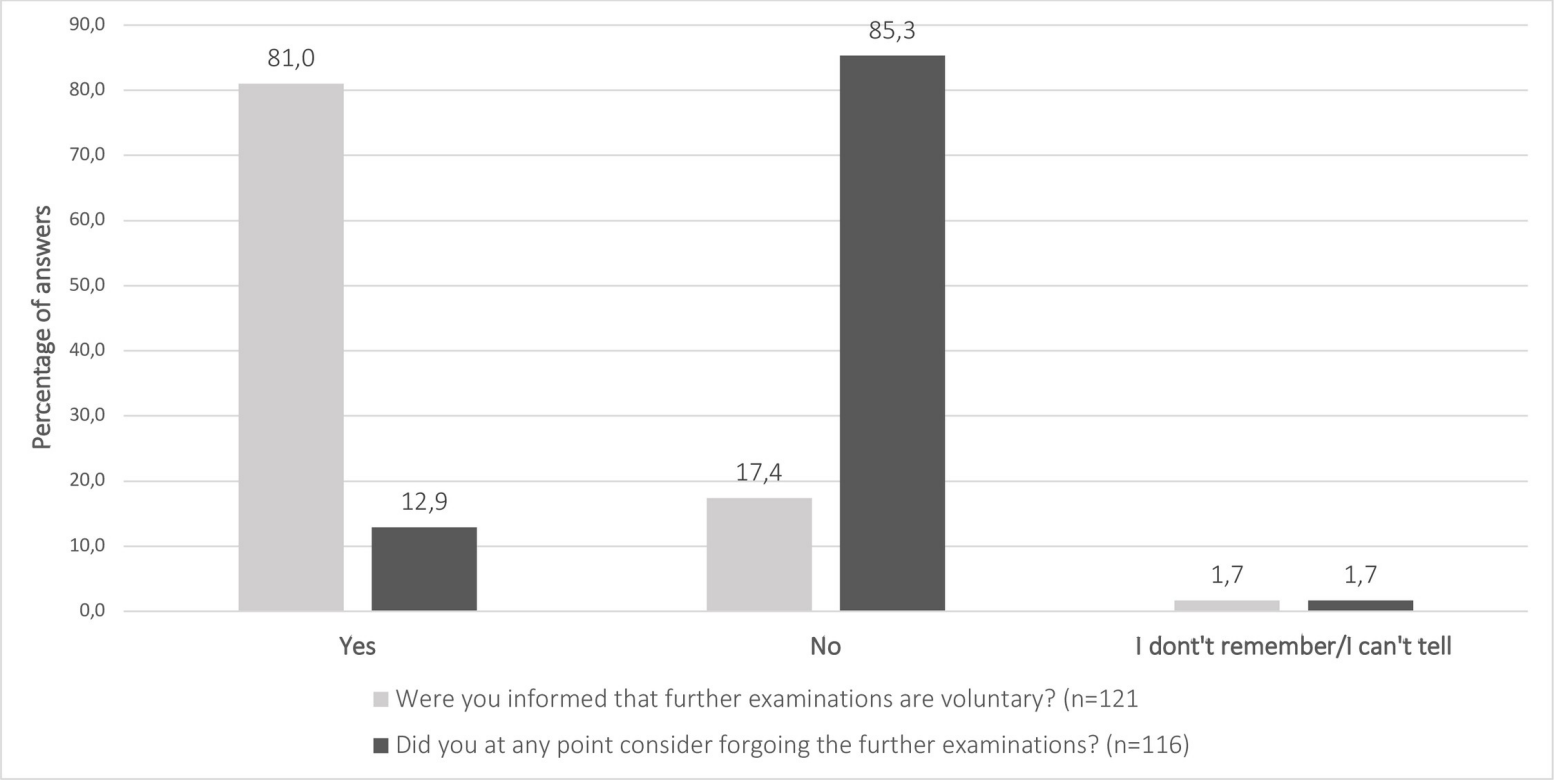
The counselling and decisions concerning optional further examinations.

Before their appointment in the FMU, 75% of women reported being either worried or very worried (4–5) about the findings of further examinations. Still only 44.5% of women had seriously considered (4–5) their personal values and options in case of a screen-positive result (Fig 4). Most women (81%) knew that further examinations are optional and most of them (85.3%) were confident about participation in further examinations (Fig 5).

**Fig 6:**
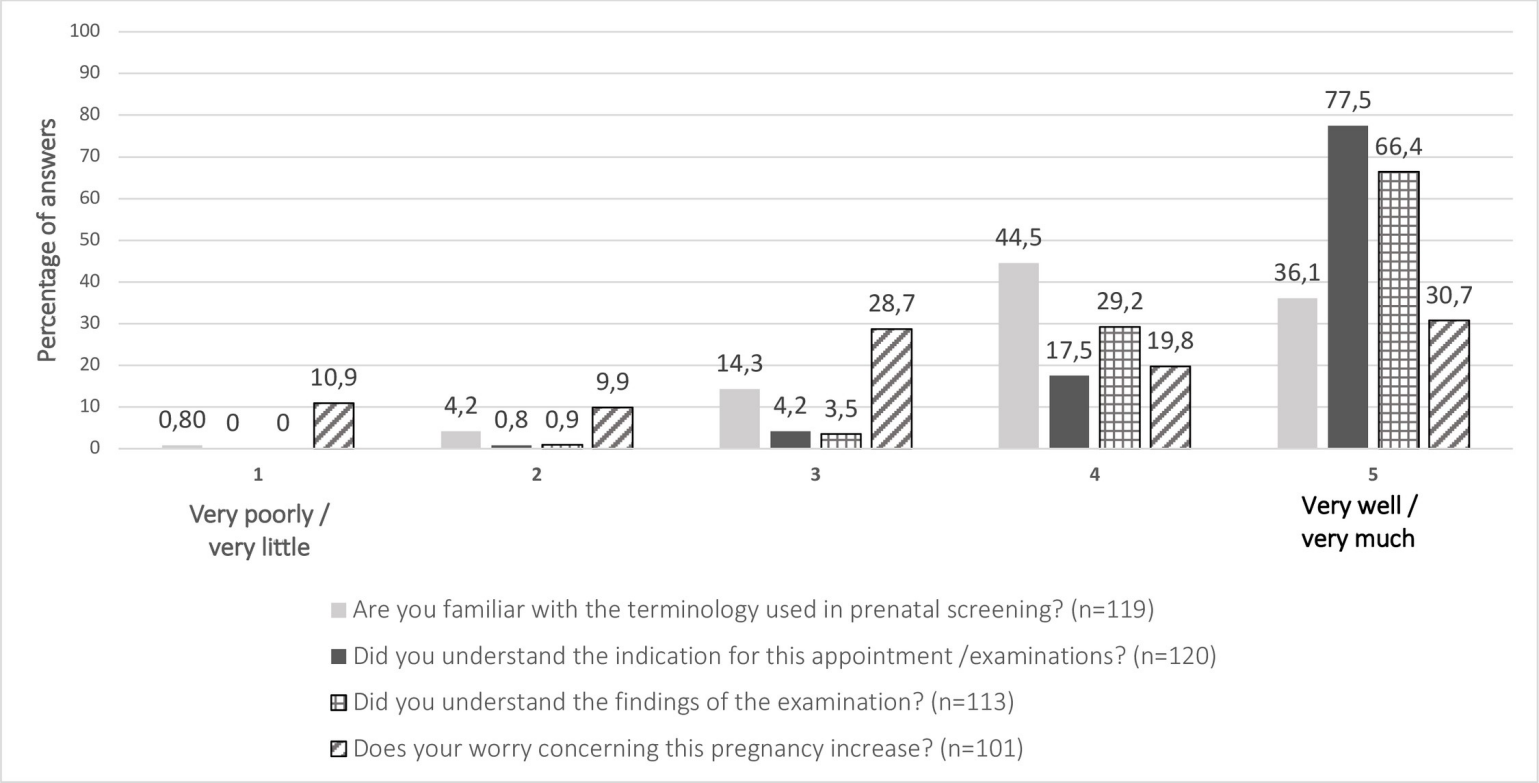
The counselling and worry concerning further examinations.

In the FMU, most (80.6%) women reported that they were familiar (4–5) with the terminology used in the FMU and the indications (95%) and the findings (95.6%) of further examinations were either well or very well (4–5) understood. Regardless of the results of the detailed US or karyotyping, 50.5% of women experienced that their worry concerning this pregnancy would increase (4–5) (Fig 6).

## Discussion

Prenatal screening is usually a positive experience for mothers-to-be and a normal result brings joy and reassurance to the expecting parents [7, 10].

A normal result in prenatal screening has been shown to decrease anxiety, make the pregnancy more real and to help the parents to bond with their child [15–18]. Problems arise, however, when an abnormal screening result is received.

As in other studies [9, 10, 19], we found that the majority experienced the upcoming examinations in the FMU with remarkable distress. Almost half of the women felt increased anxiety about their ongoing pregnancy, even if nothing abnormal was to be found in the examinations. Similarly, a study by Weinans et al. [10] found 13% of women to feel anxious even after receiving a normal result from further examinations and diagnostic tests. On the contrary, Lou et al. [15] found that even though anxiety levels peak after a positive screening result, they decrease back to normal after a negative diagnostic result, showing no evidence of residual anxiety. Since our material was collected during the appointment, we could not study this.

Our findings indicate that giving explicit and sufficient information is an area for improvement. Other studies have been conducted with similar findings, indicating that women are not as well-informed as one would hope [14, 19, 20].

Our results indicate that healthcare professionals give out good information at the time of detailed US and diagnostic tests in the FMU. The experiences about the primary counseling together with a screen-positive result are, however, less encouraging. This can, to some extent, be explained by the difficulties in receiving information at an emotionally burdened situation. Confusion and anxiety arise and due to insufficient information, many women fail to make an informed decision concerning their participance in further examinations and diagnostic tests.

In our study, some women mentioned that they had proceeded to search the internet for more information. The usage of internet as a source of information is common even though most women do not discuss this with their healthcare providers [21]. Not all information found online is reliable and finding mixed information may confuse women even more and end up increasing their anxiety.

As shown in other fields of medicine, psychological preparation is beneficial and leads to better outcomes [22]. In prenatal screening this would mean the preparation for the possibility of receiving a screen-positive result, the participation in further testing and the personal values concerning the decision of TOP beforehand. Studies conducted in Denmark and Great Britain [23, 24] support the idea of mental preparation and the importance of sufficient information, as they found that women had less decisional conflict the better informed they were. A recent study in Finland also supports the importance of informed decision, as women in late pregnancy, with a previous informed decision were more satisfied with their decisions concerning screenings [25]. Another study found well-informed women to have no more anxiety than their peers [24], but interestingly in a study by Lalor and Begley [19], women claimed that it is better not to be too informed beforehand, as extensive information could end up worrying many women for no reason.

For understandable reasons, many parents do not wish to think about the possibility of fetal abnormalities beforehand [14, 16, 17, 20] even though that is the evident reason for participating in screening. As found by Aune et al. [16], one reason for this can be that the grief is unbearable to think of in advance. According to another study from Finland, many women say they wish to make sure the baby is healthy, while knowing that the actual purpose is to find fetal anomalies [20].

The phenomenon called “optimistic bias” may play a role–parents estimate their personal likelihood of getting an abnormal screening result to be smaller than it is for other people and do not therefore feel the need to think of an adverse outcome beforehand [26].

The difficulty of understanding the risk is one of the reasons for anxiety after a screen-positive screening result. The cut-off value 1/250 (0.4%) used in combined first trimester screening is approximately the same as the risk of miscarriage after performing either the chorionic villus sample test or amniocentesis [27]. Some women could benefit from thinking about their risk the other way around. After receiving a risk value 1/20, their chance of having a healthy child is 95%. This sounds more reassuring and maybe more understandable than saying that their risk for DS is 1/20. This could help some parents on their feelings of anxiety. Some women also find the comparison between the risk of fetal abnormality and the risk of miscarriage related to invasive testing very difficult.

There have been suggestions that high stress levels and anxiety experienced during pregnancy could increase the risk of preterm delivery and interfere with maternal-fetal attachment and language development [28–32]. These suggestions together with findings concerning screening-related stress raise important ethical questions. Does the screening cause more harm than good in false positive cases [30]? Women with screen-positive results were at the time of our study offered further testing via chorionic villus sampling or amniocentesis. Today, with the non-invasive prenatal test (NIPT)

the fetal cell-free DNA can be analyzed from the mother’s blood sample. The invasive tests carry a risk for miscarriage and a chance of losing a healthy fetus [27], but the availability of NIPT has been shown to reduce the uptake of invasive testing [8].

The study by Richmond et al. reported that compared to women with low risk, those with a high- risk (≥ 1/300) result in the FTS, displayed more anxiety. They were also more likely to pursue invasive testing if NIPT was unavailable. Anxiety was reported also among women with a low-risk result in FTS, but a low-risk NIPT- result decreased anxiety in both groups to the same level [8].

NIPT is safe for the fetus and the risk of a false negative result is small [33, 34]. Still, it can be used to screen only the most common chromosomal abnormalities and by itself, is not a diagnostic method. Positive results must be confirmed via invasive methods before deciding about TOP [35, 36]. However, NIPT lessens the probability of losing a healthy fetus due to screenings and may be ethically a more appropriate method. Unfortunately, NIPT was not yet available at the time of this study. However, our focus was on counselling and the anxiety of the women. The methods of further testing can thus be seen secondary to testing methods used.

The majority of eligible women attending FMU took part in the study. Using questionnaires for data collection was convenient and gave the possibility to easily gather data from participants. The numerical scales used in most questions made analyzing the data straightforward. The open questions about the participants’ own thoughts were equally important as they gave an opportunity to express thoughts that could not be expressed in the numerical answers. There was no apparent reason for participants to give misleading answers. The reliability of result is therefore high, which can be considered as a strength of the study.

Unfortunately, some women chose not to answer all questions of the questionnaires. The influence of socioeconomical status and education was not analyzed, since this data is not systematically collected. Also, the experiences of immigrants and other minorities of population were not studied as the background and education among them has a huge variety. These can be seen as limitations of the study.

In conclusion, the counselling of health care professionals in all steps of the screening process still needs more attention and improvements. In our study, half of women did not seriously consider the possibility of getting an abnormal screening result, indicating that women do not spend a lot of time thinking their options. It is possible, that regardless of the written information and intended non-directive counselling during the first antenatal visit, the free for all prenatal screening is considered as a routine procedure that does not require serious choice-making.

High-quality counselling is essential for women to give their consent and it is of utmost importance to decrease the anxiousness and confusion concerning screening results and diagnostic examinations. Despite our high-quality antenatal care, this study suggests that we still need to improve our prescreening counselling, especially in the maternity care units and during the first antenatal visits. This is necessary to enable a real informed consent, the basic principle of screening.
